# T49. THE NEURAPRO STUDY: ADHERENCE TO STUDY MEDICATION

**DOI:** 10.1093/schbul/sby016.325

**Published:** 2018-04-01

**Authors:** Monika Schlögelhofer, Patrick D McGorry, Barnaby Nelson, Connie Markulev, Hok Pan Yuen, Miriam Schäfer, Nilufar Mossaheb, Stefan Smesny, Ian B Hickie, Gregor Berger, Eric Y H Chen, Lieuwe de Haan, Dorien H Nieman, Merete Nordentoft, Anita Riecher-Rössler, Swapna Verma, Andrew Thompson, Alison Ruth Yung, G Paul Amminger

**Affiliations:** 1 Medical University of Vienna; 2 Orygen, the National Centre of Excellence in Youth Mental Health; 3 Orygen, the National Centre of Excellence in Youth Mental Health, Medical University of Vienna; 4 University Hospital Jena; 5 University of Sydney; 6 Child and Adolescent Psychiatric Service of the Canton of Zürich; 7 University of Hong Kong; 8 Academic Medical Center; 9 Psychiatric Centre Bispebjerg; 10 Psychiatric University Clinics Basel; 11 Institute of Mental Health; 12 University of Manchester, Greater Manchester Mental Health NHS Foundation Trust; 13 Orygen, the National Centre of Excellence in Youth Mental Health, Centre for Youth Mental Health, University of Melbourne, Medical University of Vienna

## Abstract

**Background:**

Adherence to a medication is generally defined as the extent to which patients take medications as prescribed by their health care providers. Poor adherence to study medication is not uncommon posing a major challenge to treatment trails. However, poor adherence may not be randomly distributed but rather be associated with demographic or illness factors. The aim of the present study was to identify factors associated with adherence to study medication in young people at ultrahigh risk of psychosis who participated in the NEURAPRO study.

**Methods:**

Secondary analysis of data collected in a multi-centre, double-blind, placebo-controlled, randomized clinical trial to prevent or delay the onset of psychosis in participants at ultrahigh risk of psychosis testing omega-3 polyunsaturated fatty acids (omega-3 PUFAs) vs. placebo, in combination with cognitive behavioural case management (NEURAPRO) were included in this analysis. Measures included the Brief Psychiatric Rating Scale (BPRS), the Scale for the Assessment of Negative Symptoms (SANS), the Montgomery Asberg Depression Rating Scale (MADRS), the Young Mania Rating Scale (YMRS), the Social and Occupational Functioning Assessment Scale (SOFAS), and the Global Functioning: Social and Role scales. Adherence to the study medication was assessed monthly for each participant based on capsule count. The mean adherence rating over the 6-month intervention period was then computed and categorized as either adherent (≤25% of capsules returned) or non-adherent (>25% of capsules returned). Transition to psychosis was defined on the basis of operationalized criteria and assessed with the Comprehensive Assessment of the At-Risk Mental State. Levels of ω-3 PUFAs in fish oil, eicosapentaenoic acid (EPA) and docosahexaenoic acid (DHA) (amongst other fatty acids) were measured as percentage of total fatty acids in erythrocytes at baseline and at month 6 (end-of-intervention).

**Results:**

Of 304 randomised participants, 57.9% (N = 176) were non-adherent (>25% of capsules returned) and 128 (42.1%) were adherent (≤25% capsules returned) to the study medication.

No sex differences were observed for adherence rates. At baseline the omega-3 index (EPA+DHA) was significantly lower in the non-adherent group (P = 0.018). The non-adherent group had significant lower scores on the SOFAS (P = 0.001) and the Global Functioning: Social and Role Scale at baseline assessment (P < 0.001 and P = 0.020, respectively) compared to the adherent group. No statistically significant differences were observed on symptom measures at baseline (BPRS, SANS, MADRS, YMRS). The cumulative transition to psychosis rate at month 12 was significantly higher in the non-adherent group compared to the adherent group (14.8% vs. 4.7%; Log rank test: P < 0.001).

**Discussion:**

Adherence to study medication was relatively low in NEURAPRO. Poor functioning and lower levels of ω-3 PUFAs at baseline were associated with non-adherence. Young people who were non-adherent had a significantly higher risk of progressing to first episode psychosis. Knowledge about factors associated with adherence could help to improve the delivery of interventions in young people at risk of psychosis.

